# Analysis of brain network temporal reconfiguration and frequency-domain features during dynamic orchestral segmentation transition based on electroencephalography

**DOI:** 10.1371/journal.pone.0352172

**Published:** 2026-06-25

**Authors:** Yanqi Wu, Meiping Sheng, Yaping Chen, Chao Shen, Guoxun Feng

**Affiliations:** 1 School of Marine Science and Technology, Northwestern Polytechnical University, Xi’an, Shaanxi, China; 2 Ningbo Institute of Northwestern Polytechnical University, Ningbo, Zhejiang, China; 3 Ningbo Mental Hospital, Ningbo, Zhejiang, China; Federal University of Paraiba, BRAZIL

## Abstract

Background/Objectives: The human brain’s natural capacity for music perception relies on dynamic interactions across distributed neural systems. To understand this process, we investigated how the brain responds to musical segment transitions and identified the acoustic and informational features that drive these neural dynamics. Method: In a passive listening task, we used Mozart’s Serenade in G major for Strings, K.525 (Allegro) as the auditory stimulus. Based on distinct acoustic and informational profiles, two contrasting 10-s segments were selected: one transitioned from a high-frequency, less-predictable segment to a low-frequency, more-predictable segment, and the other followed the reverse pattern. We calculated metrics of EEG rhythms and brain networks and subjected them to statistical analysis to investigate their dynamics during the transitions. Results: The study reveals that the brain employs an efficiency-trade-off strategy during musical transitions. In stable periods, it conserves energy through efficient frontal and occipital processing. During a transition, the fronto-occipital-central network dynamically reconfigures, accompanied by a transient drop in global efficiency and recruitment of the right prefrontal cortex. Such resource reallocation during unpredictable shifts delays neural processing. Furthermore, *θ* power increased with greater structural complexity and a higher spectral centroid (FDR-corrected *p* < 0.05), indicating a specific mapping between these acoustic features and oscillatory activity. Our study provides evidence for the deep interactive relationship that persists between changes in musical acoustic structure and internal neural oscillations of the brain.

## 1. Introduction

Cognition emerges not from the activity of isolated brain regions, but from the dynamic interactions within and between large-scale functional networks [1,2]. These networks are not static; they undergo continuous and rapid reconfiguration to support ever-changing cognitive demands and sensory inputs [3]. Naturalistic and complex auditory stimuli, such as music, provide an ideal paradigm for studying these neural dynamics. As a universal human capacity, music perception engages a hierarchy of cognitive processes, ranging from low-level auditory feature extraction to high-level syntactic processing and emotional appreciation [4]. The temporal structure of music, replete with statistical regularities and salient boundaries, provides a unique window into how the brain parses a continuous stream of information into meaningful segments [5].

It is well-established that listening to music profoundly influences cognition and emotional regulation, thereby conferring substantial benefits to both individuals and society at large [6–9]. Yeo et al. have demonstrated that self-administered therapy training music can markedly enhance the power of *β*-waves in the bilateral prefrontal cortices, temporal lobes, and right parietal lobe. This enhancement in β-band activity is linked to higher levels of concentration and activation across a wide array of cognitive brain regions [10]. King et al., Wang et al., and Shimizu et al. found that listening to familiar music increases functional connectivity in multiple brain regions, including the default mode network (DMN), auditory, and reward networks [11–13]. However, these studies have primarily compared EEG networks across three discrete states (namely, resting state, during stimulation, and post-stimulation), and have not captured the real-time dynamics of brain activity in response to musical structure.

In research on the temporal perception of musical structure, investigations have primarily focused on duration discrimination, rhythm perception, beat extraction, and frequency perception [14,15]. Rivera-Tello studied how musical tempo affects EEG spectral dynamics [16]. Similarly, Yang et al. used music with slow and fast tempos as stimuli and elucidated the effects of music tempo on functional brain networks related to emotion regulation [17]. To investigate the effects of dissonance on the brain, Sammler et al. altered pitch within consonant musical sequences to create dissonant intervals, while keeping the overall contour structure constant [18]. Moreover, Iversen and Patel used periodic sonic pulses to study the Action Simulation for Auditory Prediction (ASAP) hypothesis [19]. Teki et al. used a sequence of clicks as stimuli and revealed that the cerebellum and the basal ganglia are part of the brain’s timing network [20]. Murdock et al. demonstrated that noninvasive 40 Hz acoustic stimulation promotes 40 Hz neural activity in multiple brain regions and attenuates pathology in mouse models of Alzheimer’s disease [21]. However, while using short, laboratory-engineered musical excerpts as stimuli allows researchers to control stimulus parameters, this approach often sacrifices musical naturalism and ecological validity, resulting in a departure from genuine musical experience.

A critical aspect of music perception is segmentation, the ability to break down the continuous flow of music into discrete perceptual units defined by segment boundaries. Musical segment boundaries are perceptually salient and theoretically well-defined, suited for studying how the brain segments naturalistic, temporally unfolding stimuli. Successful segmentation relies on the detection of acoustic and cognitive boundaries, which are often signaled by changes in harmony, rhythm, timbre, or intensity. An fMRI study by Sridharan et al. investigated the neural dynamics of event segmentation of music. By isolating time-dependent sequences of brain responses in a 10 s window surrounding section transition, the study revealed the temporal dynamics across the section transition: a ventral fronto-temporal network (VLPFC and PTC) that onsets earlier in the transition, followed in time by a dorsal fronto-parietal network (DLPFC and PPC) [22]. However, that study did not directly link these neural dynamics to specific acoustic cues such as changes in harmony or intensity.

We aim to examine two fundamental neural dimensions of dynamic music perception. First, we characterize how distinct EEG rhythms dynamically respond to structural transitions in music and how these responses are modulated by specific acoustic features. Second, we delineate the reorganization of key functional brain network metrics during shifts in musical complexity, clarifying the coupling between such network dynamics and the music’s structural attributes. We recorded EEG during a passive listening task with Mozart’s Serenade in G major, K. 525 (Allegro) [23] to examine how transitions between its segments modulate brain activity. We extracted three melodic segments and two 10-second epochs to investigate the neural dynamics during the transition between two adjacent musical segments. Given that the acoustic and informational features of musical structure are well-defined, we performed correlation analyses between these features and changes in the spectral properties of the EEG data, as well as functional brain network metrics. By elucidating these oscillatory and network reconfiguration mechanisms, we aim to provide novel insights that could inform future research on EEG-based emotion recognition and music-based cognitive interventions.

## 2. Materials and methods

**Fig 1:**
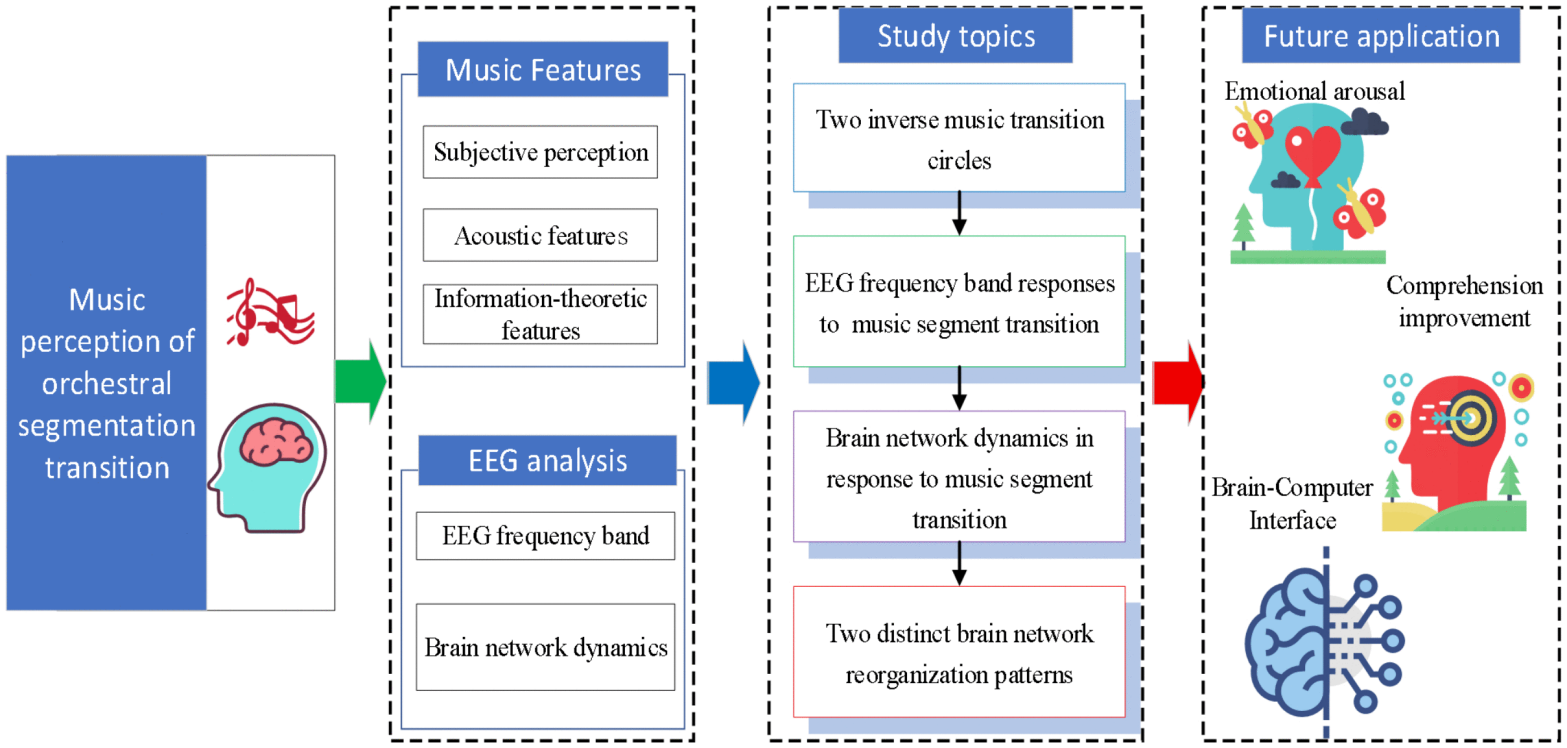
Analytical framework for brain network dynamics during orchestral transitions.

Fig. 1 outlines the study’s framework and analytical workflow. The diagram visually integrates the core components, flowing from data recording through methodology to key findings, thereby clarifying the progression and relationships between stages.

### 2.1. EEG recording

The EEG data for this study were provided by Ningbo Mental Hospital. The young adult group consisted of 36 participants (18 male/18 female; mean age = 25 ± 7 years). All participants were native Chinese speakers and had completed basic education. All had normal or corrected-to-normal vision, were naïve to formal music training, and reported no history of neurological or auditory disorders. Participants were required to avoid the following for 24 hours prior to the EEG recording: 1) consumption of nervous-system stimulants; 2) high-intensity physical or mental activities; and 3) sleep deprivation or deviation from their regular dietary patterns. All participants provided written informed consent prior to the experiment. Participant recruitment took place from 08/10/2023–01/08/2025. This study was approved by the Ethics Committee of Ningbo Mental Hospital. Written consent was obtained from all participants prior to their involvement in the study.

**Fig 2:**
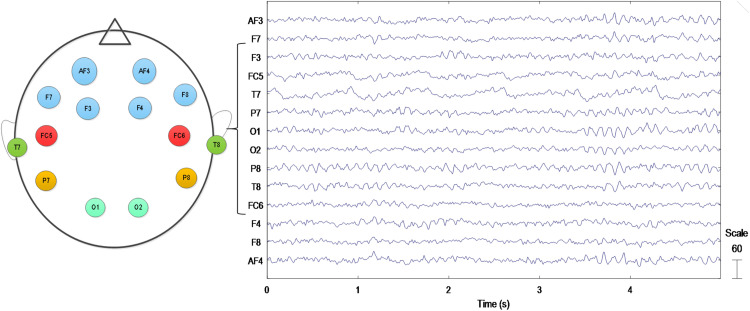
The Emotiv device electrode layout and sample EEG waveforms.

The main recording instrument was the Emotiv Epoc (Emotiv Systems, Inc.). This system acquires and processes EEG signals in real time. It features 14 non-invasive electrodes arranged according to the international 10–20 system. Data were recorded at a sampling rate of 128 Hz. The electrode layout is shown in Fig 2, along with sample waveforms. The experiment took place in a quiet, dry, dimly lit, and soundproof room to minimize interference from environmental noise and light.

### 2.2. Music stimuli analysis method

**Fig 3:**
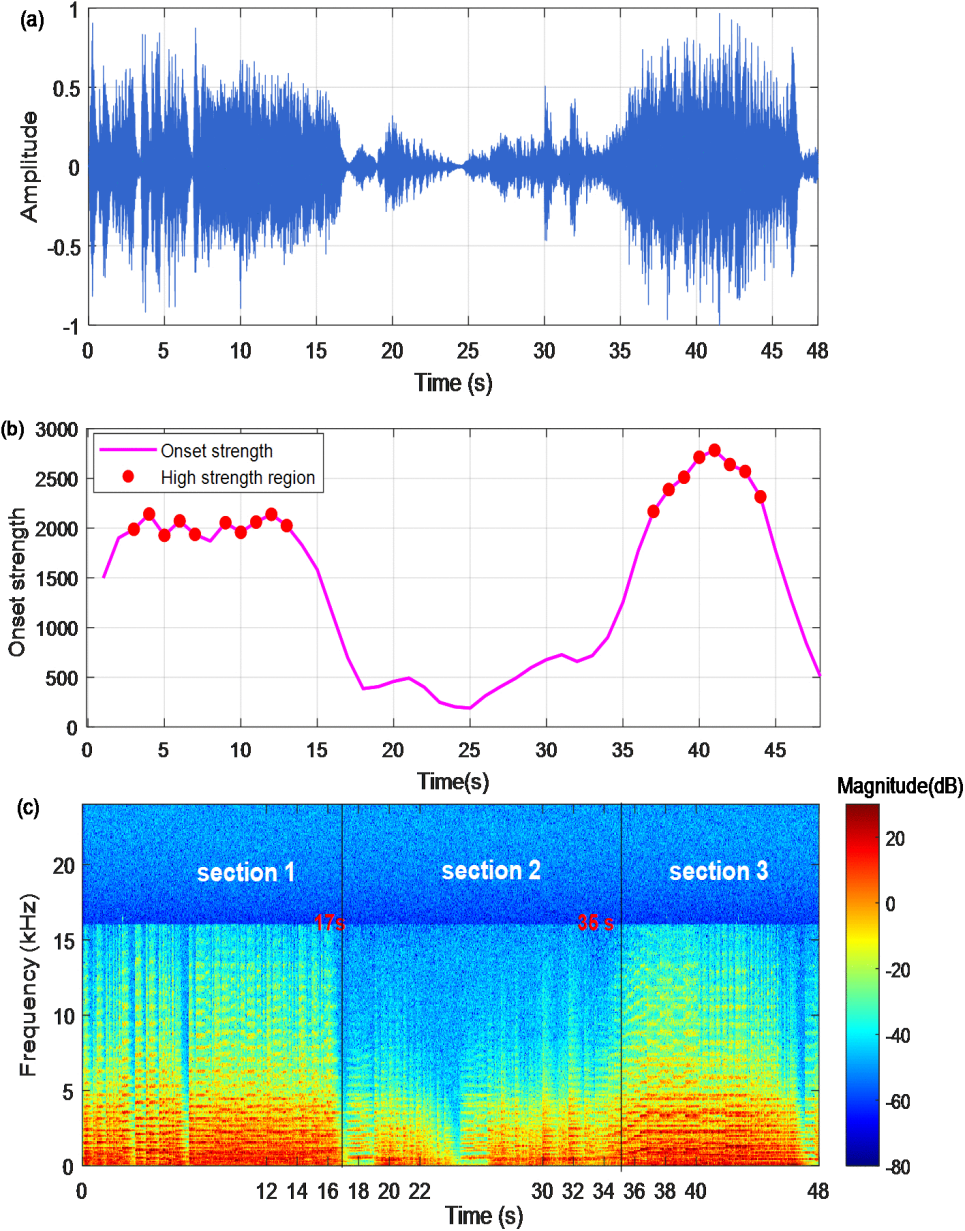
Temporal and spectral dynamics of the musical stimulus: (a) the time-domain waveform; (b) the onset strength profile; and (c) the time-frequency spectrogram.

**Fig 4:**
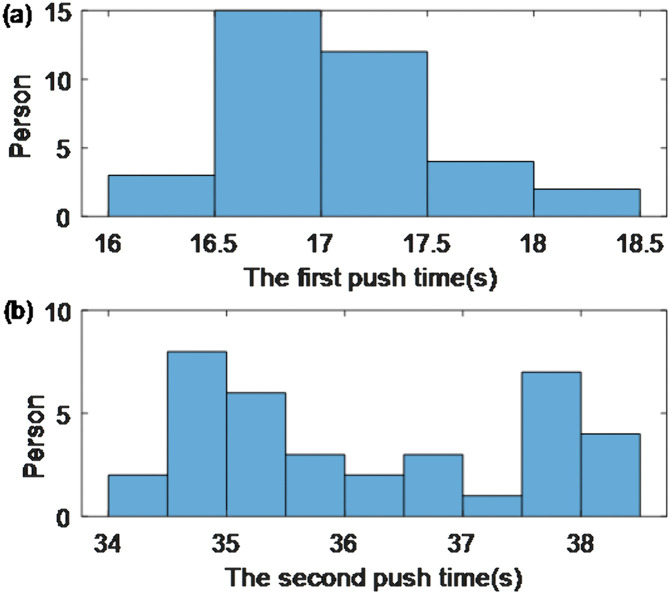
Distribution of participant response times to perceptual boundaries: (a) first transition; and (b) second transition.

We used a 48-second excerpt from the Allegro of Mozart’s Serenade in G major, K.525 as the musical stimulus [23]. Two professionally trained researchers first performed independent auditory analyses to identify perceptually salient shifts in the music, primarily based on changes in intensity, pitch, and onset strength. Onset strength serves as an acoustic feature that quantifies instantaneous energy salience in music, providing a fundamental signal for the auditory system to detect musical events and parse rhythmic structure [24,25]. To objectively render and validate these boundaries, we presented the time waveform and the onset-strength curve of the music (Fig. 3a, b). Second, we further applied a short‑time Fourier transform to confirm the precise timing of the boundaries [22]. Spectral analysis confirmed distinct frequency-band distributions: the first section (0–17 s) occupies 0–15 kHz, the second (17–35 s) is confined to 0–7 kHz, and the third (35 s onward) returns to 0–15 kHz. Centered on these two boundaries (17s and 35s), we defined successive 10-s analysis windows in 2-s steps. Participants were instructed to press a button whenever they perceived a musical transition. As shown in Fig 4, 75% of participants correctly identified the first transition (within 16.5–17.5 s), whereas only 38.89% identified the second transition (occurring at 34.5–35.5 s). Moreover, 41.67% of participants showed a 2–3 s response delay specifically for this second transition.

#### 2.2.1 Information-theoretic features.

Moreover, we extracted acoustic features and information-theoretic features from the music segments. These information features quantify the complexity level of a musical segment. In general, low complexity indicates that the observed system is highly regular, predictable structure. In contrast, high complexity corresponds to greater unpredictability and stochasticity. A variety of complexity indices have been used to analyze temporal structures in music and other domains, including entropy [26], Kolmogorov complexity [27], fractal dimension [28], and others [29]. We calculated Shannon entropy (*H*), permutation entropy (*PE*), statistical complexity (*C*_*s*_), and Kolmogorov complexity (*C*_*k*_) to assess the complexity levels of the three segments.

The audio signal was treated as a discrete random variable *X*. For *X* with *N* possible outcomes {*x*₁, *x*₂,..., *x*ₙ} and corresponding probabilities *pᵢ* = *P*(*X* = *xᵢ*), its Shannon entropy *H*(*X*) is defined as [26]:


$$\begin{array}{c} H\left(X\right)=-\sum_{i=1}^{N}p\left(x_{i}\right){log}_{b}p\left(x_{i}\right) \end{array}$$ (1)

Permutation entropy (*PE*) is a nonlinear dynamic feature used to quantify the complexity and regularity of a time series. To compute it, the phase space of the series is first reconstructed from subsequences of a chosen embedding dimension. Each subsequence is mapped to an ordinal (permutation) pattern based on the ranking of its values. The PE is then defined as the Shannon entropy of the probability distribution of these patterns [30], providing a measure of the system’s irregularity. The details of state-space reconstruction will not be elaborated here.

Statistical complexity, denoted as *C*_*s*_, quantifies the degree of structural organization within a system. It is defined as the product of shannon entropy (*H*(*P*)) and Jensen-Shannon Divergence (*D*), where *D* measures the distance between the system’s probability distribution and the uniform distribution [31].


$$\begin{array}{c} D=Q_{J}\left(P,P_{e}\right)=H\left(\frac{P+P_{e}}{\text{2}}\right)-\frac{H\left(P\right)}{\text{2}}-\frac{H\left(P_{e}\right)}{\text{2}} \end{array}$$ (2)

where *P* presents the actual probability distribution of the signal, and *P*_e_ represents the uniform distribution.

Kolmogorov complexity (*C*_*k*_) is defined as the shortest length of a computer program that can generate a target data sequence. Intuitively, more regular sequences require shorter programs, while more random ones require longer programs. In practice, it is approximated via compression algorithms. We computed it using the Lempel-Ziv (LZ) algorithm [32,33].

#### 2.2.2 Acoustic features.

We also extracted several acoustic features, including beats per minute (BPM), spectral centroid (SC), dynamic range (DR), and harmonic-to-noise ratio (HNR). The BPM was derived by detecting the periodicity in the beat-intensity envelope using an autocorrelation algorithm [34].

The formula for calculating the spectral centroid is as follows [35]:


$$\begin{array}{c} SC=\frac{\sum_{k=1}^{N}f_{k}\left|X\left(k\right)\right|}{\sum_{k=1}^{N}\left|X\left(k\right)\right|} \end{array}$$ (3)

where, *f*_k_ represents the frequency of the *k*_th_ frequency component, |*X*(*k*)| is the amplitude of the *k*_th_ frequency component, and *N* indicates the number of frequency components.

The dynamic range is defined as the ratio of the RMS of the maximum sound pressure (*p*_max,rms_) to the minimum sound pressure(*p*_min,rms_) [36]:


$$\begin{array}{c} DR=20\text{log}\left(\frac{p_{max,rms}}{p_{min,rms}}\right)\;\left(\text{dB}\right) \end{array}$$ (4)

The harmonic-to-noise ratio (HNR) quantifies the energy ratio between periodic and non-periodic components in a sound signal [36], making it a key metric for assessing signal quality.


$$\begin{array}{c} HNR=10\text{log}\left(\frac{E_{\text{harmonics}}}{E_{\text{noise}}}\right)\;\left(\text{dB}\right) \end{array}$$ (5)

where *E*_harmonic_ represents the energy of the harmonic components, and *E*_noise_ represents the energy of the noise components.

### 2.3 EEG Data analysis method

#### 2.3.1 Frequency-domain analysis.

Following the acquisition of EEG data, the initial preprocessing step involves electrode localization and re-referencing, for which we used the mean signal from the left and right mastoids as the reference for all channels [37]. Subsequently, the data were filtered using a 0.5–45 Hz band-pass filter to isolate signals of interest. Independent Component Analysis (ICA) was employed to remove ocular and muscular artifacts. Baseline correction was then applied to standardize the data. Finally, the cleaned data were subject to band-pass filtering to extract activity in the following frequency bands: delta (*δ*,1–3 Hz), theta (*θ*, 4–7 Hz), alpha (*α*, 8–13 Hz), beta (*β*, 14–30 Hz), and low-frequency gamma (*γ*_1_, 30–45 Hz) bands. This classification reflects well-documented links between oscillatory activity and distinct cognitive processes, offering a neurobiologically plausible framework for our study. Subsequently, *δ* and *θ* bands were aggregated into a slow-wave component, while *α* and *β* bands were combined into a fast-wave component. The former is chiefly implicated in internal state modulation and large-scale neural coordination, whereas the latter relates to externally oriented perceptual integration and localized cognitive processing [38,39]. Such a systemic distinction between slow and fast oscillatory systems is commonly employed in both cognitive neuroscience and clinical EEG studies [40].

The power spectrum of the frequency band was estimated using the periodogram method [41]. For a finite-length signal sequence, the periodogram is given by


$$\begin{array}{c} \hat{P}\left(\omega \right)=\frac{\text{1}}{N}\left|\sum_{n=1}^{N}x\left(n\right)e^{-j\omega n}\right| \end{array}$$ (6)

where *x*(*n*) is the *n*th sample of the sequence, *N* is the length of sequence, and *j* is is the imaginary unit.

#### 2.3.2 Brain network analysis.

Graph-theoretical analysis was employed to examine brain networks derived from EEG. Functional connections between electrode signals were first estimated, resulting in an undirected functional connectivity matrix where each element represents the synchronization strength between two electrodes. The matrix was then binarized via proportional thresholding [42]: after excluding self-connections, the top 20% of off-diagonal weights were retained, with others set to zero, producing a sparse adjacency matrix. Within this thresholded binary network, the local efficiency of information processing was quantified using the clustering coefficient *C*. For a node *i*, *C* is calculated as the ratio of the actual number of edges between its neighbors (*M*_*i-neighbors*_) to the maximum possible number of such edges (*M*_*i-connected*_) [43]:


$$\begin{array}{c} C\left(i\right)=\frac{M_{i-connected}}{M_{i-neighbors}} \end{array}$$ (7)

where *C*(*i*) ranges from 0 to 1. Values of 0 and 1 respectively correspond to the absence of any connections among the node’s neighbors and the presence of all possible connections, forming a fully connected clique.

The characteristic path length (*L*) of a network is defined as the average of the shortest paths between all node pairs [44], that is,


$$\begin{array}{c} L=\frac{\text{1}}{M(M-1)}\sum_{i\neq j}l_{ij} \end{array}$$ (8)

where, *M* represents the number of nodes in the network, and *l*_*ij*_ represents the shortest path length between nodes *i* and *j*. The characteristic path length *L* reflects the overall efficiency of information integration across different brain regions, rather than the information processing efficiency of local brain regions.

As a more robust metric that naturally handles disconnected nodes, global efficiency is defined as the average inverse shortest path length [45],


$$\begin{array}{c} Eglob=\frac{\text{1}}{M(M-1)}\sum_{i\neq j}\frac{\text{1}}{l_{ij}} \end{array}$$ (9)

Degree centrality quantifies the number of direct connections a node has within a network [46]. Nodes with high degree centrality have numerous connections to other brain regions and typically act as central hubs for information exchange,


$$\begin{array}{c} DC\left(i\right)=\frac{\text{1}}{M-1}\sum_{i\neq j}a_{ij} \end{array}$$ (10)

where, the element *a*_*ij*_ in the network connectivity matrix represents the connection between nodes *i* and node *j*.

## 3. Results

### 3.1. Characterization of acoustic and information-theoretic features

**Fig 5:**
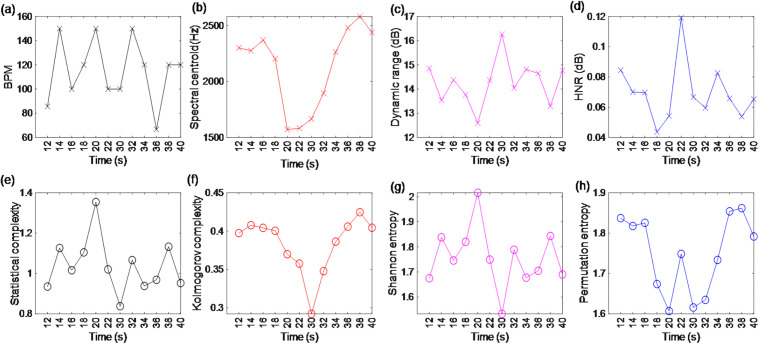
Acoustic features and information-theoretic features of the music stimuli: (a) BPM; (b) spectral centroid; (c) dynamic range; (d) HNR; (e) statistical complexity; (f) Kolmogorov complexity; (g) Shannon entropy; and (h) permutation entropy.

Fig 5 illustrates the temporal evolution of acoustic features and information-theoretic metrics. The analysis spans two observation windows covering one complete transition cycle: from a high-frequency, high-complexity segment to a low-frequency, low-complexity segment, and back again. BPM fluctuated between 100 and 150 throughout. The spectral centroid (SC) mirrored the spectrogram, decreasing and then increasing in frequency. Both dynamic range (DR) and the harmonic-to-noise ratio (HNR) reached their minima between 18 and 20 seconds, then recovered and entered a period of fluctuation. Although the two complexity metrics showed some discrepancies, both reached their lowest values around the 30-second mark. Kolmogorov complexity was lower in central regions and higher at boundaries, aligning with the SC trend. Statistical complexity and Shannon entropy were relatively higher during the 12–22 s interval than during 30–40 s. Permutation entropy was consistently lower in the second segment compared to the others.

### 3.2. EEG frequency band responses to music segment transition

#### 3.2.1. EEG frequency band power analysis.

**Fig 6:**
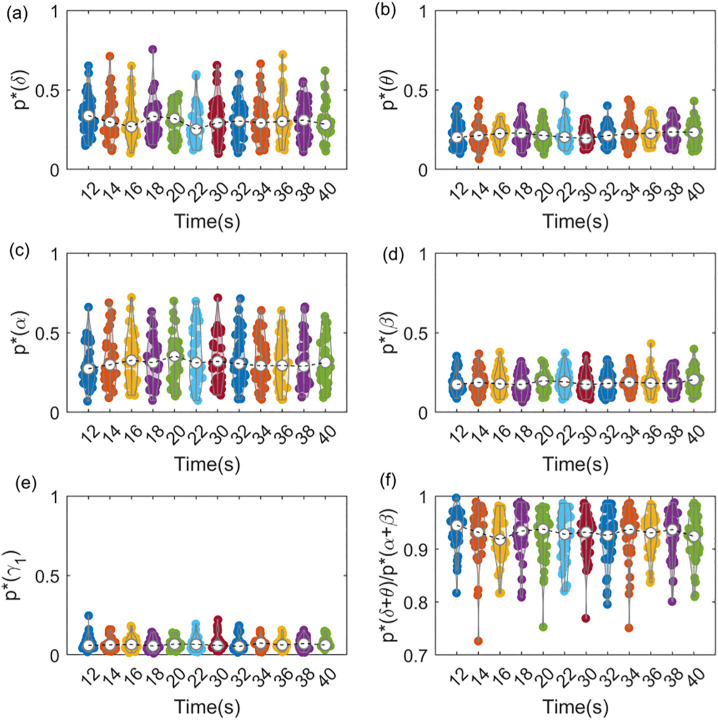
The variation of different band power during two transition circles: (a) *δ* wave; (b) *θ* wave; (c) *α* wave; (d) *β* wave; (e) *γ*_1_ wave; and (f) the ratio of *p*(*δ* + *θ*) to *p*(*α* + *β*).

At each time point, the power spectral density (PSD) of the EEG was computed. The absolute power for each frequency band was obtained by integrating the PSD over its specific range. To control for inter-participant baseline differences, absolute band power was normalized by the total power, yielding relative power values between 0 and 1. The time courses of these relative powers for the *δ*, *θ*, *α*, *β*, and *γ*_1_ bands were shown in Fig 6(a)–(e). Additionally, Fig 6(f) presented slow-to-fast wave power ratio.

Across all frequency bands, the lower-frequency oscillations (*δ*, *θ*, and *α*) exhibited the most significant modulations, while the high-frequency *β* and *γ*_1_ bands remained relatively stable. Notably the brain’s response was time-dependent, differing substantially between the first transition (from high-frequency, high-complexity to low-frequency, low-complexity music) and the second transition (back to high-frequency, high-complexity music). The *δ* wave exhibited a triphasic pattern (decrease–increase–decrease) during the first transition but was minimal during the second transition. *θ* power showed a brief rise and fall in the first transition, contrasting with a more sustained increase in the second. The *α* band increased then decreased in the first transition, with negligible change in the second. Given that the *β* band is linked to sustained attention, and the *γ*_1_ band to feature binding and high-level cognition, their stability suggests that the core perceptual and cognitive engagement required by the music remained consistent across transitions. The slow-to-fast wave power ratio exhibited a triphasic pattern during the first transition, whereas it remained stable throughout the second transition. The opposing dynamics of *δ* decrease and *α* increase during the transition, which reversed afterward, suggest a coordinated mechanism for shifting cognitive states.

#### 3.2.2 Correlations between EEG bands and musical features.

**Fig 7:**
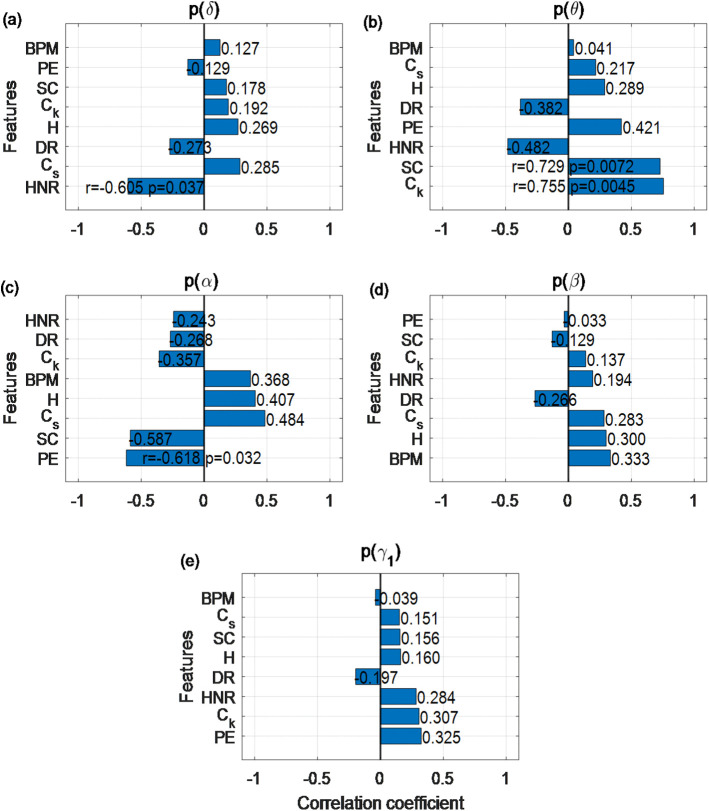
Ranking of correlation coefficients between musical features and (a) *δ* wave power; (b) *θ* wave power; (c) *α* wave power; (d) *β* wave power; (e) *γ*_1_ wave power.

**Fig 8:**
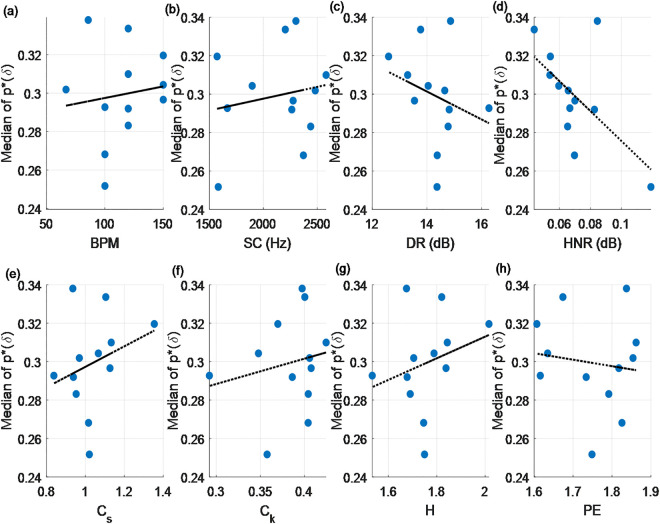
Scatter plots and fitted regression curves between *δ* band power and music features, such as (a) BPM; (b) SC; (c) DR; (d) HNR(*); (e) C_s_; (f) C_k_; (g) *H*; and (h) PE.

**Fig 9:**
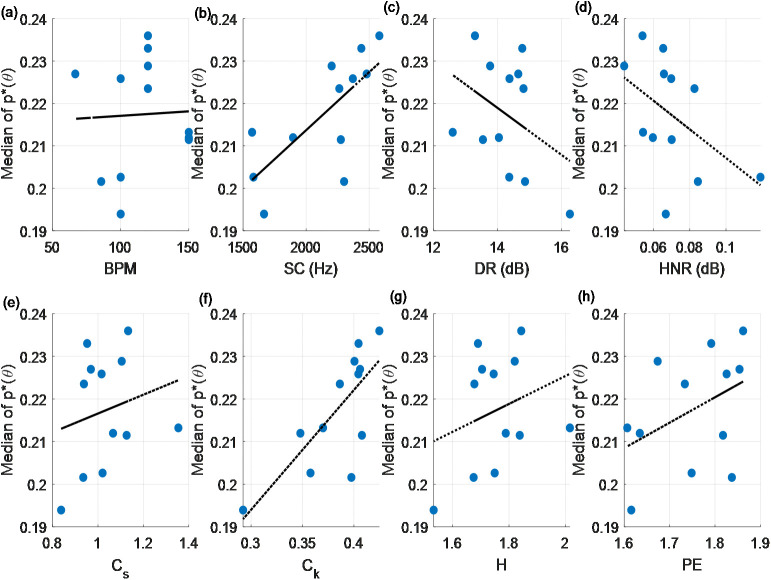
Scatter plots and fitted regression curves between *θ* band power and music features, such as (a) BPM; (b) SC (*); (c) DR; (d) HNR; (e) *C*_s_; (f) *C*_k_ (*); (g) *H*; and (h) PE.

**Fig 10:**
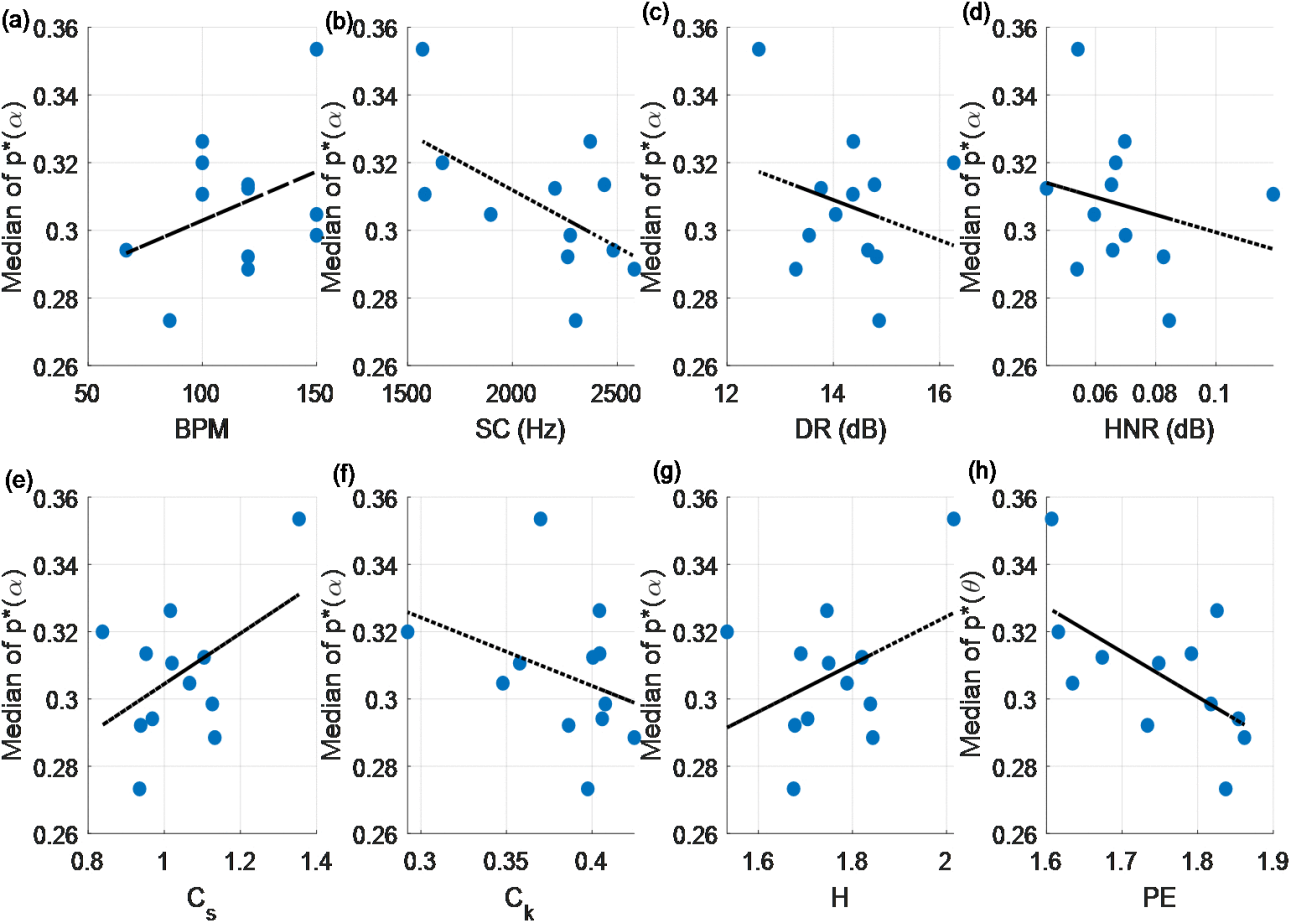
Scatter plots and fitted regression curves between *α* band power and music features, such as (a) BPM; (b) SC (*); (c) DR; (d) HNR; (e) *C*_s_; (f) *C*_k_; (g) *H*; and (h) PE (*).

To examine the relationship between oscillatory brain activity and musical acoustic features, we performed Pearson correlation analyses between the power of EEG frequency bands (*δ*, *θ*, *α*, *β*, *γ*_1_) and eight musical features. To control for multiple comparisons while retaining exploratory sensitivity, a two-stage false discovery rate (FDR) correction [47] was applied: first within each frequency band (as an independent family), and then globally across all tests. The correlation results, sorted in ascending order of the coefficient values, are shown in Fig 7. Figs 8–10 further display scatter plots with fitted regression curves for the relationships between music features and *δ*, *θ*, and *α* bands, respectively. Table 1 shows the uncorrected *p*-values, the family-wise FDR-adjusted *p* (F_FDR *p*), and the global FDR-adjusted *p* (G_FDR *p*).

**Table 1:** Results of correlation analysis before and after FDR correction.

Power	Statistics	Features of music stimuli							
		BPM	SC	DR	HNR	Cs	Ck	H	PE
*δ*	*r*	0.127	0.178	−0.273	−0.605	0.285	0.192	0.269	−0.129
	*p*	0.693	0.580	0.391	0.037	0.370	0.549	0.397	0.688
	G_FDR *p*	1.000	1.000	1.000	0.515	1.000	1.000	1.000	1.000
	F_ FDR *p*	1.000	1.000	1.000	0.296	1.000	1.000	1.000	1.000
*θ*	*r*	0.041	0.729	−0.382	−0.482	0.217	0.755	0.289	0.421
	*p*	0.899	0.007	0.221	0.112	0.498	0.005	0.363	0.173
	G_FDR *p*	1.000	0.216	1.000	0.890	1.000	0.216	1.000	1.000
	F_ FDR *p*	0.899	0.036	0.353	0.230	0.569	0.036	0.484	0.345
*α*	*r*	0.368	−0.587	−0.273	−0.243	0.484	−0.357	0.407	−0.618
	*p*	0.239	0.045	0.399	0.447	0.111	0.254	0.189	0.032
	G_FDR *p*	1.000	0.515	1.000	1.000	0.889	1.000	1.000	0.515
	F_ FDR *p*	0.382	0.258	0.456	0.456	0.296	0.382	0.379	0.258
*β*	*r*	0.333	−0.129	−0.266	0.194	0.283	0.137	0.300	−0.033
	*p*	0.290	0.688	0.403	0.546	0.372	0.671	0.343	0.918
	G_FDR *p*	1.000	1.000	1.000	1.000	1.000	1.000	1.000	1.000
	F_ FDR *p*	1.000	1.000	1.000	1.000	1.000	1.000	1.000	1.000
*γ*_1_	*r*	−0.039	0.156	−0.197	0.284	0.151	0.307	0.160	0.325
	*p*	0.904	0.629	0.540	0.370	0.639	0.331	0.619	0.303
	G_FDR *p*	1.000	1.000	1.000	1.000	1.000	1.000	1.000	1.000
	F_ FDR *p*	1.000	1.000	1.000	1.000	1.000	1.000	1.000	1.000

As summarized in Fig. 7, correlations between *δ* power and most acoustic features were either negligible (|*r*| < 0.2 for BPM, PE, SC, *C*ₖ) or weak (|*r*| ≈ 0.2–0.3 for H, DR, *C*ₛ). A notable exception was a moderate negative correlation with Harmonic-to-Noise Ratio (HNR; *r*=−0.606, *p* = 0.037), which did not survive family-wise FDR correction (F_FDR *p* = 0.296). As shown in the scatter plot in Fig. 8, HNR exhibits an inverse relationship with *δ* power. In contrast, *θ* power showed strong positive correlations with Cₖ (*r* = 0.755, *p* = 0.0045) and SC (*r* = 0.729, *p* = 0.0072). Both correlations remained significant after family‑wise FDR correction (F_FDR *p* = 0.036). Associations with HNR (*r* = −0.482) and PE (*r* = 0.421) were weaker and nonsignificant. *α* power was negatively correlated with PE (*r* = −0.618, *p* = 0.032) and SC (*r* = −0.587, *p* = 0.045). This indicates that *α* activity was suppressed in response to increases in both entropy and spectral centroid (Fig. 10). Correlations with *C*ₛ, *H*, and BPM were weak and non‑significant. However, none of these *α*‑band associations survived family‑wise FDR correction. No significant linear correlations were observed between *β* or *γ*1 power with any acoustic feature, either before or after multiple comparison correction, indicating that these high-frequency oscillations were not systematically modulated by the selected musical features under the present paradigm.

To assess the robustness of the findings, a global FDR correction was applied across all statistical tests as an additional, more stringent control. Under this conservative criterion, none of the correlations between EEG band powers and musical features remained statistically significant (all G-FDR *p* > 0.05). Consequently, when requiring associations to withstand the strictest study‑wide error control, no universally robust linear correlation pattern can be concluded.

Nevertheless, within the family-wise FDR correction, suggestive trends was observed. By integrating these trends into the established neurophysiological functions of each frequency band, we propose an exploratory framework for future validation. *θ*-band showed the most robust associations. After family-wise FDR correction, *θ* power remained significantly correlated with both C*ₖ* and SC (F-FDR *p* < 0.05). This suggests that greater musical structural complexity and higher spectral energy may enhance *θ* synchronization, potentially underpinning deeper cognitive processing and emotional immersion. Although the correlations for the *δ* and *α* bands did not survive multiple-comparison correction, their effects were theoretically valuable. The reduction in *δ* activity with higher HNR is a plausible neurophysiological marker of arousal and exogenous attention. Conversely, suppression of *α* band activity in response to harsh or unpredictable sounds could signal an adaptive transition toward heightened alertness and psychophysiological tension.

### 3.3 Brain Network Dynamics in response to music segment transition

#### 3.3.1 Local clustering coefficients.

**Fig 11:**
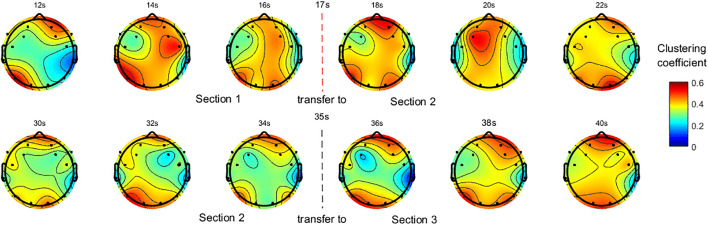
Clustering coefficients during two transitions.

We first analyzed the local clustering coefficient, quantifying the connection density among a node’s neighbors as an indicator of localized processing efficiency. Fig. 11 displays its dynamics during the two transitions.

As revealed in Fig 11, the brain networks exhibited dynamic collaboration among the prefrontal cortex (PFC, cognitive control network), the occipital lobe (imagery generation network), and central area (sensorimotor network) in response to musical transitions. During the first musical transition, the right frontal cortex and left occipital lobe were activated initially to cope with the sudden change. From 14 s to 18 s, connectivity expanded to right frontal-central areas, suggesting a shift in cognitive demand from initial detection to sustained execution. A similar pattern were observed from 38 s to 40 s of the second transition. Notably, between 20 s to 22 s, elevated activity in the left frontal lobe indicated a shift in cognitive demand toward structural analysis and internal association. In contrast, during the second transition (30 s-36 s), local connection weakened, and activity become more concentrated in the prefrontal and occipital lobes. This reflected a more focused allocation of resources to the core cognitive control (prefrontal) and internal imagery generation (occipital) networks. Furthermore, from 38 s to 40 s, the regions with the highest clustering coefficients expanded to include the right frontal-central areas, though the overall connection strength was lower than during the 14–18 s period. The delayed shift in regions with high clustering coefficients during the second transition aligns with the behavioral observation that participants identified this boundary with a 2–3 s delay. This correspondence suggests that the slower network reconfiguration may underlie the postponed perceptual recognition. Behaviorally, responses were faster for transitions from high-frequency, unpredictable to low-frequency, predictable segments than for transitions in the opposite direction.

#### 3.3.2 Global network metrics.

**Fig 12:**
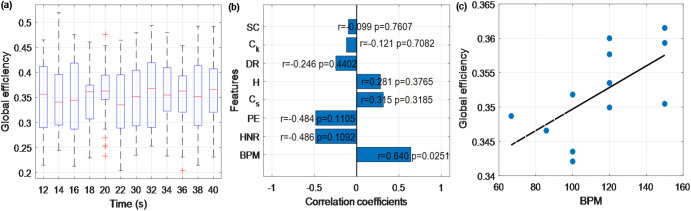
Temporal dynamics of the global efficiency and its correlation with musical features during two transition periods: (a) time-course variation, (b) correlation coefficients, and (c) a scatter plot of global efficiency versus BPM.

Fig 12 illustrates the dynamics of global efficiency and its correlation with musical features during two transition periods. Overall, the global efficiency varied between 0.2 and 0.5, with the group median indicated by the red line in Fig 12(a). The whiskers of the box plot suggest a trend of initial decrease followed by an increase during both transitions. During the first transition, the median value decreased initially, then increased, and finally decreased again. In contrast, throughout the second transition, the median value exhibited continuous fluctuation. The correlations between musical features and global efficiency are depicted in Fig 12(b), where r and p denote the correlation coefficient and its uncorrected significance level, respectively. The analysis revealed that musical tempo (BPM) had the strongest association (r = 0.640, uncorrected p = 0.025), as illustrated in the scatter plot (Fig 12(c)). However, this correlation did not survive FDR correction (adjusted p = 0.201).

**Fig 13:**
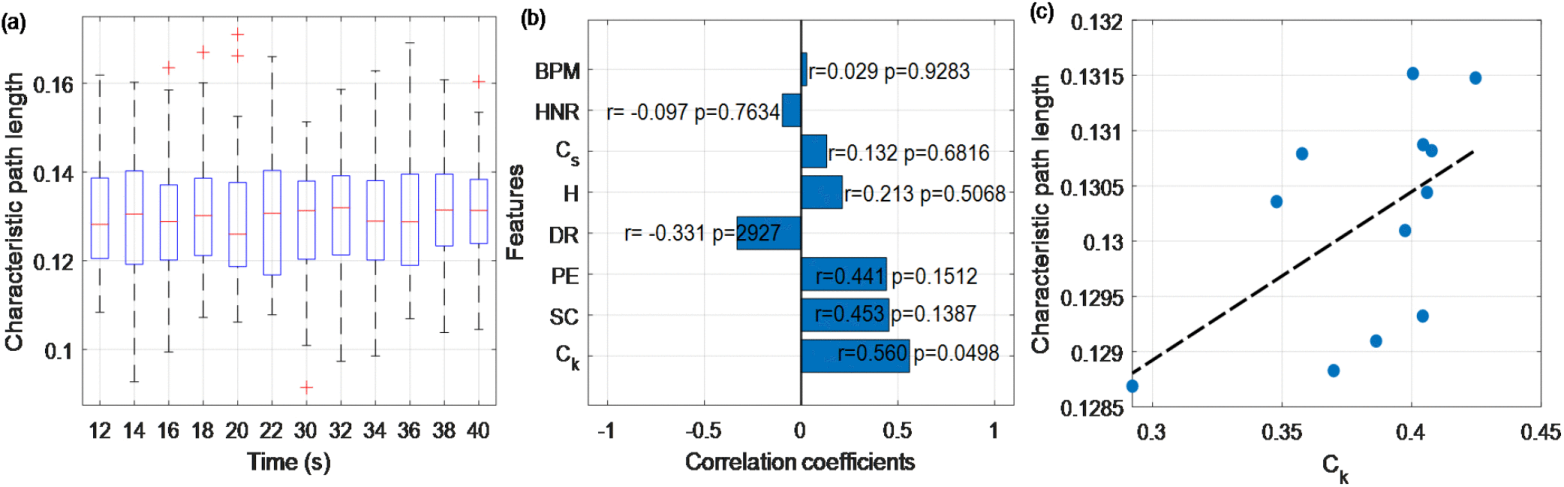
Temporal dynamics of the characteristic path length and its correlation with musical features during two transition periods: (a) time-course variation, (b) correlation coefficients, and (c) a scatter plot of characteristic path length versus C_k_.

Fig 13 illustrates the dynamics of the characteristic path length and its correlation with musical features across two transition periods. The overall path length varied between 0.1 and 0.17, with the group median indicated by the red line in Fig 13(a). The whiskers of the box plot suggested a trend of an initial increase followed by a decrease during the transitions. This trend in path length is inversely related to that of global efficiency, consistent with their mathematical definitions. Correlations with musical features are summarized in Fig 13(b). Kolmogorov complexity (C_k_) showed the strongest association with path length (r = 0.560, uncorrected p = 0.0251), indicating that higher complexity was associated with a longer characteristic path length. However this association did not survive FDR correction (adjusted p = 0.3984). Collectively, the results of Fig 12 and Fig 13 may suggest that different musical features appear to selectively influence distinct aspects of the brain network.

During a musical transition, the heightened clustering from the prefrontal and occipital lobes expands into frontal-central areas, accompanied by an increase in characteristic path length and a decrease in global efficiency. Once the transition is resolved, the frontal-central areas are released, allowing global efficiency to recover. Frontal networks exhibit clear task-dependent lateralization: complex music biases processing toward the right prefrontal cortex for novelty detection and global integration, whereas simpler music biases processing toward the left prefrontal cortex for structural analysis and internal association. Collectively, these findings indicate that the brain adapts to musical-structural changes via an efficient strategy, dynamically balancing local segregation with global integration and flexibly engaging lateralized frontal lobe.

To identify hub electrodes that demonstrated sustained importance throughout the entire task period, we performed the following analysis. First, for each of the 35 participants across 12 time points, we counted how often each electrode ranked among the top K in degree centrality (K = 1–5). Second, a permutation‑based test was used to assess whether these observed frequencies exceeded chance level [48]. Under the null hypothesis (equal selection probability for all electrodes), 2000 permutations were generated to create a null distribution. The standardized residual (Z‑score) between the observed frequency and the expected frequency was then computed for each electrode. Electrodes with |Z| > 1.96 (two‑tailed p < 0.05) were considered to exhibit statistically sustained importance. The results are summarized in Table 2.

**Table 2:** Distribution test of hub electrode frequency and significant electrodes across different *K.*

*K Electrodes*	Test of electrode selection frequency vs. uniform distribution			Details of Significant Electrodes		
	G_obs	G_perm	*p*-value	Electrode (s)	Uncorrected *p*	FDR *p*
1	0.471	0.096 ± 0.0194	<0.001	AF3	<0.001	<0.001
2	0.404	0.0650 ± 0.0130	<0.001	AF3	<0.001	<0.001
				F7	<0.001	<0.001
				FC5	0.002	0.003
3	0.324	0.050 ± 0.010	<0.001	AF3	<0.001	<0.001
				F7	<0.001	<0.001
				FC5	0.001	0.0017
4	0.269	0.042 ± 0.008	<0.001	AF3	<0.001	<0.001
				F7	<0.001	<0.001
				F3	0.006	0.009
				FC5	0.008	0.010
				T7	0.008	0.010
5	0.219	0.036 ± 0.007	<0.001	AF3	<0.001	<0.001
				F7	<0.001	<0.001
				F3	0.008	0.012
				FC5	0.015	0.020
				T7	0.002	0.005

For all K values (1–5), the Gini coefficient of the observed network significantly exceeded that of the random expectation (p < 0.05), leading to the rejection of the null hypothesis of equal importance across electrodes. FDR-corrected tests identified a set of electrodes whose importance was significantly above chance. Electrodes AF3 and F7 showed robust significance (FDR-corrected p < 0.001); electrode FC5 was consistently significant for K ≥ 2; electrodes F3 and T7 were incorporated into the significant set when K ≥ 4. To most comprehensively capture the core hub network, all five significant electrodes identified under the K = 5 condition (AF3, F7, F3, FC5, T7) were carried forward for subsequent analysis.

**Fig 14:**
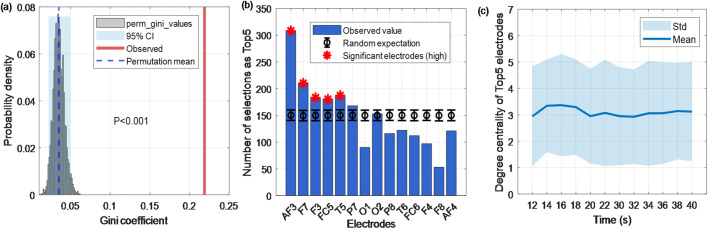
Hub identification and dynamics across two musical transitions: (a) Gini coefficient comparison between the observed network and random networks; (b) frequency distribution; and (c) time course of hub degree centrality.

Fig 14 shows the dynamics of degree centrality across transition periods. For K = 5, the Gini coefficient of the random networks followed a normal distribution, with values concentrated in the range of 0–0.05, indicating a relatively uniform distribution of nodal importance under the null model. In contrast, the observed network exhibited a significantly higher Gini coefficient of 0.22, demonstrating that hub importance within the actual brain network was highly concentrated. The frequency distribution of electrodes for K = 5 is shown in Fig 14(b). Electrode AF3 occurred most frequently, followed by F7. The frequencies of F3, FC5, and T7 were comparable to each other, yet all were significantly higher than the random expectation. Fig 14(c) displays the temporal trajectories of degree centrality for these five hub electrodes. Analysis revealed a distinct temporal pattern: during the first transition period, degree centrality showed an overall increasing trend, suggesting a progressive strengthening of hub function. In contrast, the curves remained relatively flat throughout the second transition period.

## 4. Discussion

By analyzing time-varying EEG responses to transitions between auditory segments, this study investigates the specific associations between musical acoustic features and brain networks. Our analyses reveal two key findings: first, the brain’s response to dynamic musical transitions is characterized by high temporal precision and stimulus-specific asymmetric network reorganization; second, some acoustic features modulated three key frequency bands: *θ* power was enhanced by musical complexity and spectral energy (surviving multiple‑comparison correction), whereas modulations of *α* and *δ* power, while observable, did not survive rigorous correction and thus require more cautious interpretation.

AF3, F3, F7, FC5, T7 collectively delineate a classic left fronto-temporal pathway, serving as strategic sites for information convergence and distribution during auditory processing. Built upon this core set of hub connections, the brain network reconfigured in different ways during the two transition periods.

Transition 1 (From low to high predictability): This shift was marked by an initial rise in local connectivity followed by a decline, accompanied by a lateralization shift from the right to the left prefrontal cortex. This pattern suggests that the brain first rapidly mobilizes neural resources, engaging cognitive control (right prefrontal cortex), visual imagery (occipital lobe), and sensorimotor integration (central regions) to process the unpredictable input. As the new segment became predictable, processing stabilized into a more efficient mode centered on the left prefrontal cortex for structural analysis.

Transition 2 (From high to low predictability): In contrast, when predictability decreased, the network maintained an energy-conserving state governed by predictive coding. Neural resources were not broadly redeployed until the prediction error (driven by increasing complexity and unpredictability) surpassed a threshold, at which point cognitive integration was initiated. This comparison also shows that the transition from predictable to unpredictable segments leads to prolonged neural processing and reaction times, as the brain engages in iterative refinements driven by prediction error [49].

Furthermore, our findings reveal a robust enhancement of *θ* oscillations in response to complex musical structures and brighter timbres, which survived multiple‑comparison correction. This provides direct electrophysiological evidence for the engagement of demanding cognitive processes, including continuous memory updating, structural prediction, and affective evaluation, consistent with prior work identifying frontal *θ* as a signature of cognitive control and deep engagement with music [50]. In contrast, modulations in the *δ* and *α* bands, while suggestive, did not survive rigorous correction and thus must be interpreted with greater caution. For *δ* activity, higher HNR was associated with reduced *δ* power. Given that *δ* activity is often associated with states of sensory disengagement (e.g., deep sleep), this may tentatively suggest that acoustically pure, information‑dense music prompts a shift away from low‑arousal states toward higher‑arousal processing. According to the inhibitory gating framework [51], *α* oscillations serve as a functional inhibitory mechanism, suppressing cortical excitability to guide attention and information flow. This framework offers a plausible account for the observed *α* power dynamics: during simple, predictable segments, elevated *α* power likely reflects the efficient suppression of task‑irrelevant or predictable auditory input, optimizing resource allocation. Conversely, when music becomes complex, the rapid reduction in *α* power may disinhibit auditory regions, thereby facilitating detailed sensory processing of the unexpected or information-rich content. Our research precisely links these oscillatory dynamics to both informational and acoustic features of music, demonstrating that both structural unpredictability and perceptual saliency effectively drive functional activation of the brain. The main limitation is the relatively fixed nature of the observation window and music samples. Future research could: (1) adopt longer musical stimuli and more ecological listening paradigms; (2) explore the relationship between a wider range of musical features (such as tonality and rhythmic complexity) and neural responses; and (3) integrate technologies with higher spatial resolution such as fMRI to more accurately localize the neural sources of these oscillatory changes.

## 5. Conclusion

By precisely regulating the acoustic characteristics of music and synchronously recording EEG activity, this study systematically elucidates the dynamic neural mechanisms underlying the brain’s response to musical stimuli. Key findings demonstrate that the brain’s response to musical transitions is highly adaptive. Even given two contrasting transition types, we observed persistent neural response differences, indicating that processing is not linear but shaped by prior experience, expectation, and plasticity. Analysis of brain network efficiency and local connection reorganization further confirm that the brain adapts optimal strategies to cope with the challenges of musical information flow transitions by flexibly adjusting the patterns of coordination and integration between internal brain regions. This study not only verifies that music is a powerful tool capable of regulating brain states in multiple dimensions, but more importantly, it provides a precise mechanistic explanation for this regulation from the perspective of neural oscillations: music interacts with the brain’s inherent rhythmic activity systems through its acoustic properties, thereby achieving fine-grained regulation of cognitive states, emotional arousal levels, and information processing modes. These findings hold important implications for fields such as music therapy, brain-computer interface development, and artificial intelligence. Building on this foundation, future research could further explore more complex musical paradigms and the mechanisms by which their long-term effects influence neural plasticity.
